# Mitogen-Activated Protein Kinase Inhibitors and T-Cell-Dependent Immunotherapy in Cancer

**DOI:** 10.3390/ph13010009

**Published:** 2020-01-07

**Authors:** Sandeep Kumar, Daniel R. Principe, Sunil Kumar Singh, Navin Viswakarma, Gautam Sondarva, Basabi Rana, Ajay Rana

**Affiliations:** 1 Department of Surgery, Division of Surgical Oncology, University of Illinois at Chicago, IL 60612, USA; ksandeep@uic.edu (S.K.); principe.danny@gmail.com (D.R.P.); singhsk@uic.edu (S.K.S.); navinv@uic.edu (N.V.); sondarva@uic.edu (G.S.); basrana@uic.edu (B.R.); 2 Jesse Brown VA Medical Center, Chicago, IL 60612, USA; 3 Medical Scientist Training Program, University of Illinois College of Medicine, Chicago, IL 60612, USA; 4 University of Illinois Hospital & Health Sciences System Cancer Center, University of Illinois at Chicago, Chicago, IL 60612, USA

**Keywords:** cancer, mitogen-activated protein kinase, T cells, Programmed cell death protein 1, Programmed death-ligand 1, cytotoxic T-lymphocyte-associated protein 4, T-cell anergy, immunotherapy

## Abstract

Mitogen-activated protein kinase (MAPK) signaling networks serve to regulate a wide range of physiologic and cancer-associated cell processes. For instance, a variety of oncogenic mutations often lead to hyperactivation of MAPK signaling, thereby enhancing tumor cell proliferation and disease progression. As such, several components of the MAPK signaling network have been proposed as viable targets for cancer therapy. However, the contributions of MAPK signaling extend well beyond the tumor cells, and several MAPK effectors have been identified as key mediators of the tumor microenvironment (TME), particularly with respect to the local immune infiltrate. In fact, a blockade of various MAPK signals has been suggested to fundamentally alter the interaction between tumor cells and T lymphocytes and have been suggested a potential adjuvant to immune checkpoint inhibition in the clinic. Therefore, in this review article, we discuss the various mechanisms through which MAPK family members contribute to T-cell biology, as well as circumstances in which MAPK inhibition may potentiate or limit cancer immunotherapy.

## 1. Introduction

Mitogen-activated protein kinase (MAPK) signaling is mediated by several MAPK family members, sharing several evolutionary-conserved domains [1]. Together, these events are contributing to a wide range of cellular function including proliferation [2], migration [3], angiogenesis [4], invasion [5], metastasis [6] and apoptosis [7]. Classically, MAPK signals are activated downstream of receptor tyrosine kinases, including epithelial growth factor receptor (EGFR) [8]. However, in cancer, MAPK signaling is commonly hyperactivated due to gain of function mutations in proto-oncogenes including B-Raf proto-oncogene, serine/threonine kinase (B-Raf) [9], neuroblastoma RAS viral (v-ras) oncogene homolog (NRAS) [10], Kirsten rat sarcoma viral oncogene homolog (KRAS) [11], Raf-1 proto-oncogene, serine/threonine kinase (RAF1) [12], or loss of function mutations to negative regulators including neurofibromatosis type 1 (NF1), in each case leading to enhanced cell proliferation and survival [13].

As such, MAPK signaling generally promotes tumor growth and various MAPK family members have been proposed as candidates for therapy. Such approaches have shown promising results in both in preclinical studies and in clinical trials [14]. Though encouraging, the global effects of MAPK inhibition within the tumor microenvironment (TME) are poorly understood. Given the advent of cancer immunotherapy, which is now first-line therapy in several solid malignancies, it is essential to better evaluate the effects of MAPK inhibition on local immune function.

Previous reports suggest that MAPK signaling is essential for T-cell development [15], activation [16], proliferation and survival [17]. Unsurprisingly, MAPK signaling is also implicated in directing interactions between tumor cells and the surrounding T-cell infiltrate, though these roles are complex and often contradictory. For instance, MAPK signaling has been shown to suppress the expression of negative immune checkpoints such as programmed death-ligand 1 (PD-L1) and cytotoxic T-lymphocyte-associated protein 4 (CTLA-4) in several cancers [18]. Similarly, various MAPK members down regulate T-cell costimulatory molecules such as tumor necrosis factor receptor superfamily, member 4 (TNFRSF4), also known as CD134 or OX40 and tumor necrosis factor receptor superfamily member 9 (TNFRSF9) also known as CD137 or 4-1BB, thereby impeding T-cell activation and effector function [19]. Therefore, therapeutic inhibition of various MAPK family members has been proposed as a potential means to augment immune checkpoint inhibitors. Here, we discuss about the current generations of MAPK inhibitors targeting mitogen-activated protein kinase kinase/extracellular signal-regulated protein kinases (MEK/ERK), c-Jun N-terminal kinases (JNK), and p38 mitogen-activated protein kinases (p38 MAPK), as well as the means through which they may cooperate with cancer immunotherapy.

## 2. MEK/ERK Inhibition

ERK was the first MAPK family member to be cloned and characterized [20], and is most commonly activated by the upstream RAS/RAF/MEK cascade [21]. ERK signaling regulates a variety of benign and malignant cell functions, including proliferation, differentiation, motility, and survival [22]. While the role of ERK signaling is well described in tumor cells, ERK is also crucial in the regulation of several aspects of T-cell biology, including positive/negative selection in the thymus [23]. In mature T-cells, ERK is activated following interaction between the T-cell receptor (TCR) and major histocompatibility complex (MHC) on an antigen-presenting cell [24], where it functions to direct the activation of a T cell [25] as well as interleukin-2 (IL-2) production and clonal expansion [26]. This is particularly true with respect to effector CD8^+^ T-cells, which are dependent on ERK signaling to remain functionally active [27].

Several selective inhibitors of ERK signaling are reported to have marked antitumor efficacy, including FR180204 [28], BVD523 [29], CC90003 [30], GDC-0994 [31] and MK-8353 [32]. BVD523 (Ulixertinib) specifically has been used in clinical trials, showing clear efficacy in patients who have been previously treated with immunotherapy [29].

Mitogen-activated protein kinase kinase (MEK, also known as MAP2K) is an upstream MAPK kinase family member that phosphorylates MAPK members ERK, p38 and JNK [33], though MEK is most clearly associated with ERK activation [34]. Accordingly, MEK/ERK has also been proposed as a potential target for therapy (Table 1). Several MEK inhibitors have been reported including Trametinib [35], Cobimetinib [36], Binimetinib [37], Selumetinib [38], some of these have been investigated in clinical trials. Specifically, Trametinib has been used with 4-1BB and OX40 agonists to enhance T-cell costimulation [19]. Early results suggest that this combined approach induces robust antitumor effects mediated predominantly by cytotoxic T lymphocytes [19]. Additionally, Selumetinib also appears to attenuate PD-L1 expression without impeding interferon gamma (IFNγ)-induced MHC-I upregulation in nonsmall cell lung cancer cells, suggesting that it may also benefit patients receiving cancer immunotherapy [39]. The MEK inhibitor G-38963, along with anti-PD-L1 therapy promotes effector function and lifespan of tumor-infiltrating CD8^+^ T-cells leading to synergistic inhibition in tumor growth [40]. While exciting, the efficacy of such combinations are in early stages and MEK/ERK inhibitors have yet to be fully explored in combination with immune checkpoint inhibition. However, given the known functions of ERK in impeding antitumor immune responses and promising results of early trials, this approach warrants continued consideration.

## 3. JNK Inhibition

JNK proteins are also MAPK family members and were first discovered in the 1990s [41]. JNK signals are activated by several upstream MAPK members [42] as well as G-protein-coupled receptors (GPCRs) [43]. Like other MAPKs, JNK signaling pathways regulate several cellular functions [44]. Accordingly, JNK signals also appear to regulate T-cell differentiation and function [45], though the contributions of JNK signaling are re-multifaceted and context-dependent. For example, when thymocytes undergo apoptosis, JNK2 targets Jun proto-oncogene (c-JUN) to promote cell death, whereas during proliferation, JNK2 targets nuclear factor of activated T-cells (NFAT) to mediate DNA binding [46]. Further, JNK cooperates with MEKK2 signaling to direct IL-2 biosynthesis, serving crucial roles in TCR/CD3-mediated T-cell signaling [47]. Interestingly, JNK appears to differentially regulate CD4^+^ and CD8^+^ T-cell function [48]. For example, loss of JNK1 enhances IL-2 production and proliferation in CD8^+^ T-cells, though this was not observed in CD4^+^ T-cells [48]. However, there is a current lack of consensus over the dominant role of JNK in T-cell immunity, and the effects of broadly administered JNK inhibitors on tumor infiltrating lymphocytes are not well studied.

However, there are several emerging JNK inhibitors, including SP600125 [49], AS601245 [50], CC-401 [51], AS602801 [52], D-JNKI-1 [53] and BI-78D3 [54], that are now showing early efficacy in various solid tumor types [55]. Interestingly, the JNK inhibitor SP600125 has been suggested to rescue cytotoxic T-lymphocytes from activation-induced cell death without diminishing their capacity to synthesize cytotoxic cytokines such as IFNγ [56]. Hence, while JNK is not yet substantiated as a viable target for combined immunotherapies, it warrants future consideration in combination with immune checkpoint inhibitors.

## 4. p38 MAPK Inhibition

The p38, another MAPK family member, also appears to have important roles in immune function. The p38 MAPK is activated by environmental stress as well as several inflammatory cytokines [57]. Like other MAPKs, the functions of p38 have been well-established in tumor cells, directing any number of cell processes including differentiation, migration, and inflammation [58]. Accordingly, p38 overexpression is associated with poor responses to conventional therapy in several cancers including breast cancer, nasopharyngeal carcinoma, gastric, and pancreatic cancer [59,60,61,62].

While these events are well understood, the role of p38 in T-cell function is less clear and also highly context-specific. The p38 MAPK appears to regulate synthesis of a wide range of proinflammatory cytokines including tumor necrosis factor (TNF) [63] and interleukin-17 (IL-17) [64]. Additionally, p38 regulates T-cell activation via selective activation of NFATc [65], itself an important mediator of CD8^+^ T-cell effector function [66]. However, p38 also has important roles in regulatory T-cells (Tregs), namely those induced in the tumor microenvironment. These Tregs function largely to suppress autoreactive T-cells, thereby limiting the effects of tumor immunotherapy [67]. As p38 has been shown to promote Treg-mediated immune suppression [67], therapeutic inhibition of p38 MAPK may benefit patients receiving T-cell-based immunotherapies.

To date, a number of selective inhibitors of p38 MAPK have been synthesized including SCIO-469 [68], BIRB-796 [69], LY2228820 [70], VX-745 [71], SB203580 [72] and PH-797804 [73]. Several are beginning to show early efficacy in cervical [74], pancreatic, ovarian, [75] and breast cancers [70]. Additionally, p38 MAPK inhibition cooperated with anti-CD137 to promote antitumor T-cell responses in glioblastoma [76]. Further, p38 inhibition appears to increase the expression of OX40L and downstream OX40 signaling in dendritic cells (DCs), thereby potentiating effector T-cell function [77]. Hence, while the clinical utility of p38 inhibition also remains unclear, p38 inhibitors also warrant exploration in the setting of immune checkpoint inhibition.

## 5. Other MAPK Family Members

Several nonclassic MAPK family members also appear to have paramount roles in T-cell biology, and their inhibition may also warrant consideration as an adjuvant to immunotherapy. For example, Hematopoietic Progenitor Kinase-1 (HPK1) is a member of the MAP4K family expressed largely in hematopoietic cells [78], and acts upstream of MAPK signaling [79]. HPK1 has been reported to regulate both nuclear factor kappa-light-chain-enhancer of activated B cells (NFkB) and activates JNK signaling pathway [80], and is associated with progression of gastrointestinal tumors [81]. The roles of HPK1 in T-cell function is now becoming clear. For instance, studies using a model of autoimmune encephalomyelitis suggest that HPK1 negatively regulates T-cell activation and function [82]. Similarly, in in vivo models of lung cancer, the loss of HPK1 enhances the effector function of cytotoxic T-cells [83]. However, the translational relevance of these findings is unclear at this time.

Another upstream regulator of MAPK signaling, germinal center-like kinase (GLK or MAP4K3) belongs to serine/threonine-protein kinase, Ste20 family of protein kinases [84], and may also have important roles in T-cell function. GLK is activated by stimuli including ultraviolet radiation and several proinflammatory cytokines [84], and has clear roles in the pathogenesis of both autoimmune disease and cancer [85]. Clinically, GLK overexpression predicts poor overall and progression-free survival in non-small-cell lung carcinoma (NSCLC) [86], though the mechanisms through which GLK contributes to disease pathogenesis are not clear at this time. However, emerging evidence suggests that GLK regulates T-cell activation via IL-17A signaling [87]. Accordingly, the GLK inhibitor, Verteporfin, has been approved by FDA in macular degeneration of eyes [88] and may warrant exploration in cancer.

## 6. Future Perspective

MAPK signaling has been implicated in cancer incidence and progression for decades, contributing to nearly all hallmark features of tumorigenesis. Accordingly, physicians and scientists alike have long been eager to introduce MAPK inhibitors to the clinical management of cancer patients. Several MAPK family inhibitors have shown substantial efficacy in clinical trial, with some earning FDA approval. While these results are no doubt encouraging, MAPK signals have important roles in a variety of cell functions extending well beyond the cancer epithelium and, as described above, direct any number of immunologic processes.

**Figure 1:**
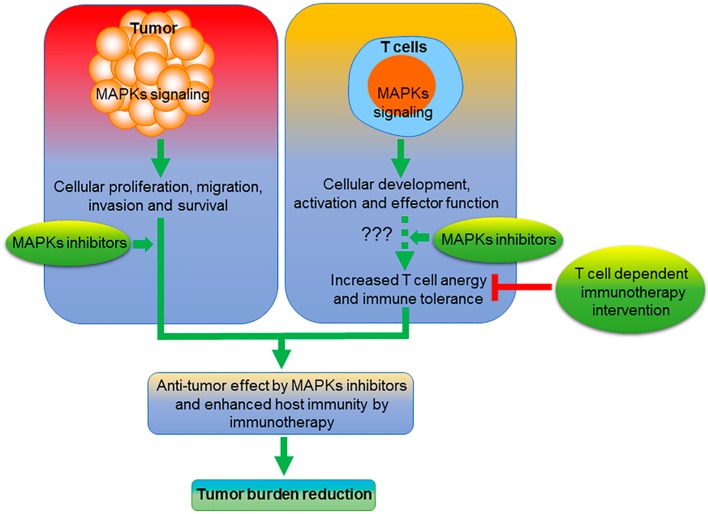
Schema describing the potential interaction between MAPK inhibitors and cancer immunotherapy. In the proposed model, we suggest that MAPK inhibition may function through two distinct mechanisms. While blockade of various MAPKs limits the proliferation of tumor cells and promotes apoptosis, they may also precipitate T-cell exhaustion and/or anergy, which may potentially be reversed through the use of selective immunotherapies.

**Figure 2:**
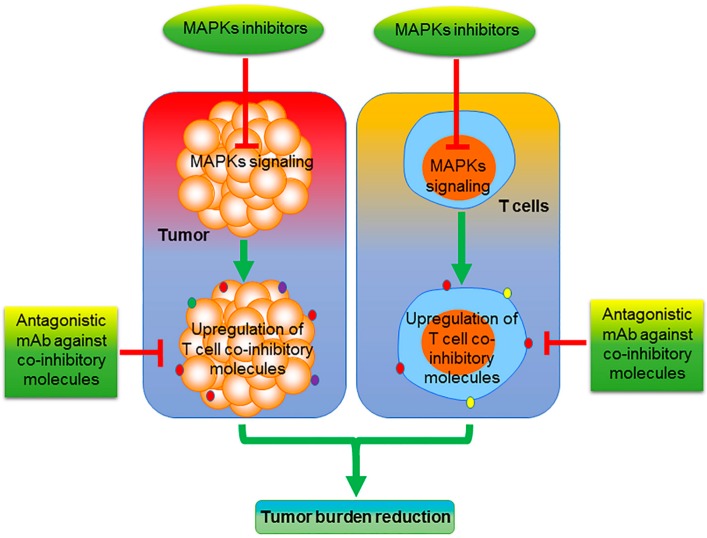
Schema describing the potential induction of T-cell coinhibitory molecules as an unintended consequence of MAPK inhibition. In the proposed model, we suggest that MAPK inhibition may lead to the unintended upregulation of coinhibitory, immune checkpoint molecules on the surface of cancer and T cells alike, which may facilitate tumor escape from immune surveillance. mAb, monoclonal antibody.

While selective MAPK inhibitors may potentiate cancer immunotherapy, due to the important roles of MAPKs in T-cell function, in some instances, MAPK inhibition may have unintended consequences with respect to cancer immunotherapy (Figure 1). Hence, the success of combining cancer immunotherapy with MAPK inhibitors may be highly context-dependent, and requires further study before advancing such strategies to the clinic. While neutralizing antibodies against PD-1 or PD-L1 are the most frequently used in cancer therapy and may cooperate with MAPK inhibitors (Figure 2), there are several other alternate approaches that are now emerging that have not been adequately evaluated in the background of MAPK inhibition.

While we have discussed recent efforts involving OX40 and 4-1BB, other immune checkpoints are now under early investigation including the myeloid cell-intrinsic immune-checkpoint V-domain immunoglobulin suppressor of T-cell activation (VISTA), though it is unknown how strategies targeting VISTA will interact with MAPK inhibition [89]. Further, as chimeric antigen receptor T (CAR-T) cell therapy advances into clinical use for acute lymphoblastic leukemia and advanced lymphomas [90], the combination of CAR-T-cells and MAPK inhibitors also warrants exploration. However, regardless of the approach, given the complex and often contradictory roles of MAPKs in tumor immunology, MAPK inhibitors may potentiate or limit the efficacy of cancer immunotherapy, and their addition to any immune regimen warrants careful consideration. The role of MAPK inhibitors on T-cell function is controversial and could have differential impact in in vitro and in vivo setups. Even in in vivo condition, the effects of MAPK inhibitors on tumor infiltrating T-cells depend on several factors, including TME. However, some of the in vivo results demonstrated a robust antitumor efficacy with the combination of MAPK inhibitors along with T-cell-dependent immunotherapy, suggesting that the future of MAPK inhibitors and immunotherapy is promising.

## Figures and Tables

**Table 1:** MEK/ERK inhibitors and immunotherapy in cancer.

MEK/ERK Member	Inhibitor	Combination with Immunotherapy	Cancer Type
MEK1/2	Trametinib	4-1BB and OX40 agonist antibodies	Breast cancer [19]
	Selumetinib	Anti-EGFR antibody	Lung adenocarcinoma [39]
	G-38963	Anti-PD-L1 antibody	Colon carcinoma [40]
ERK1/2	BVD523	Positive outcomes in patients previously treated with immunotherapy	NRAS-, BRAF V600–, and non–V600 BRAF-mutant solid tumors [29]
